# Supplementary material for: Modulation of Synthetic Tracheal Grafts with Extracellular Matrix Coatings
Source: Bioengineering (Basel). 2021 Aug 20;8(8):116. doi: 10.3390/bioengineering8080116 (PMC8389233; doi:10.3390/bioengineering8080116)
Supplement: Supplementary file 1 [file bioengineering-08-00116-s001.zip › bioengineering-1264691-supplementary.pdf]

Supplementary data of:

Modulation of Synthetic Tracheal Grafts with Extracellular Matrix Coatings

Lumei Liu [1,2], Sayali Dharmadhikari [1,2], Robert A. Pouliot [3], Michael M. Li [2], Peter M. Minneci [1], Zhenghong Tan [4], Kimberly Shontz [1], Jed Johnson [5], Susan D. Reynolds [6], Christopher K. Breuer [1,7], Daniel J. Weiss [2] and Tendy Chiang [1,2,*]

[1] Center of Regenerative Medicine, Abigail Wexner Research Institute, Nationwide Children's Hospital, Columbus, OH 43215, USA; lumei.liu@nationwidechildrens.org (L.L.); Sayali.Dharmadhikari@nationwidechildrens.org (S.D.); pmminneci@gmail.com (P.M.M.); Kim.Shontz@nationwidechildrens.org (K.S.); christopher.breuer@nationwidechildrens.org (C.K.B.)

[2] Department of Pediatric Otolaryngology, Nationwide Children's Hospital, Columbus, OH 43205, USA; Michael.Li@osumc.edu

[3] Department of Medicine, Larner College of Medicine, University of Vermont, Burlington, VT 05405, USA; Robert.Pouliot@uvm.edu (R.A.P.); daniel.weiss@med.uvm.edu (D.J.W.)

[4] College of Medicine, The Ohio State University, Columbus, OH 43210, USA; ZhengHong.Tan@osumc.edu

[5] Nanofiber Solutions, Inc, Columbus, OH 43017, USA; jed.johnson@nanofibersolutions.com

[6] Center for Perinatal Research, Abigail Wexner Research Institute, Nationwide Children's Hospital, Columbus, OH 43215, USA; Susan.Reynolds@nationwidechildrens.org

[7] Department of Pediatric Surgery, Nationwide Children's Hospital, Columbus, OH 43205, USA

[7]

* Correspondence: Tendy.Chiang@nationwidechildrens.org; Tel.: +1-(614)-722-6600, Fax: +1-(614)-722-6609

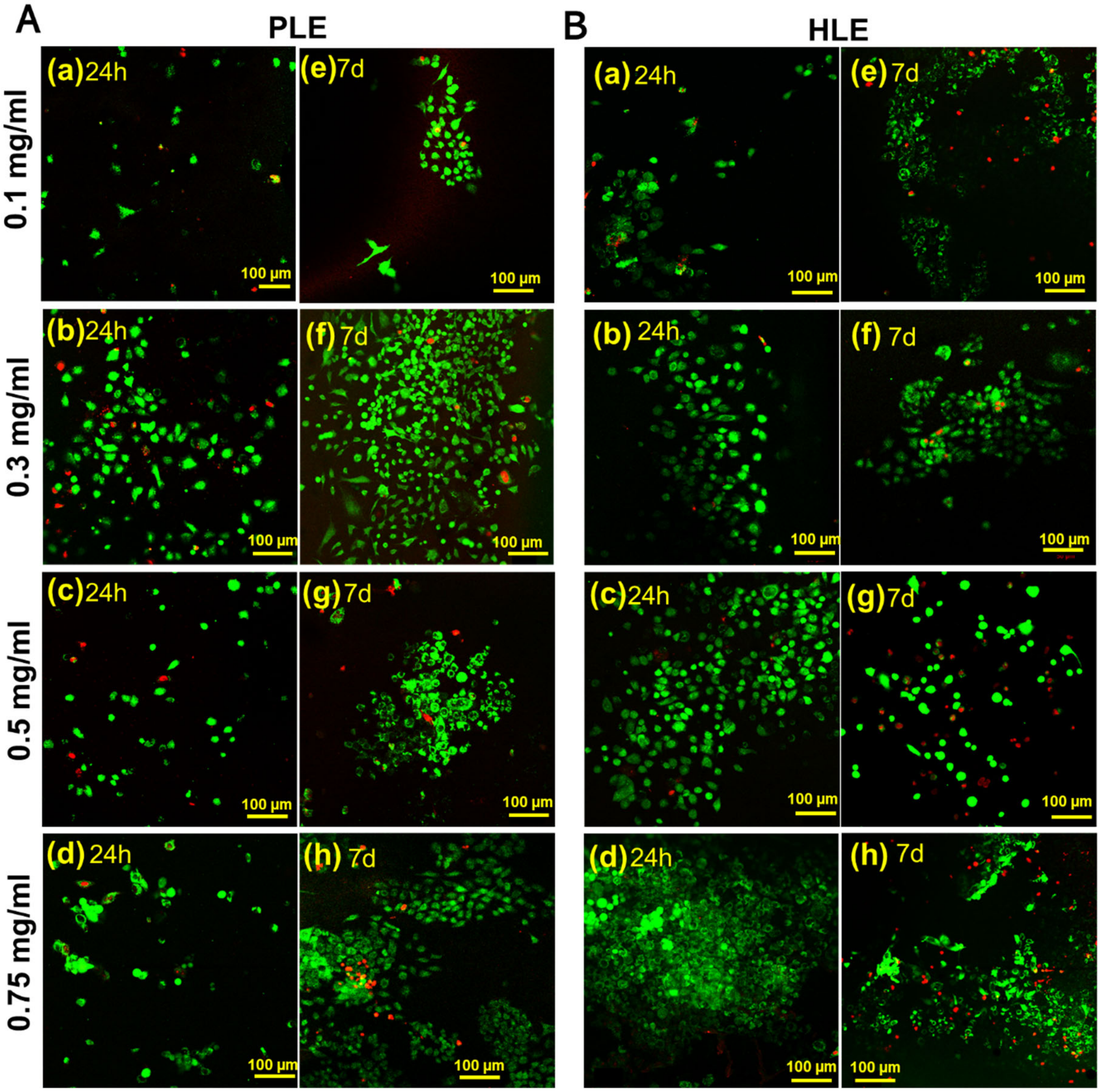

Figure S1. Representative live/dead images of (A) cells on PLE-coated scaffolds for 24h ((a)~(d)) and 7d ((e)~(f)), and (B) cells on HLE-coated scaffolds for 24h ((a)~(d)) and 7d ((e)~(f)). Live cells are stained in green and red cells are stained in red.
